# Sleep Quality, Daytime Napping, and Dream Recall in Older Adults: A Biopsychosocial Study of Health, Loneliness, and Humor Styles

**DOI:** 10.7759/cureus.107940

**Published:** 2026-04-29

**Authors:** Agni Nakou, Elena Dragioti, Stefanos Mantzoukas, Christos Tsironis, Eleni Bartzou, Fotios Tatsis, Nektaria Zagorianakou, Mary Gouva

**Affiliations:** 1 Scientific Laboratory of Psychology and Person-Centered Care, School of Health Sciences, University of Ioannina, Ioannina, GRC; 2 Research Laboratory of Integrated Health, Care and Well-Being, School of Health Sciences, University of Ioannina, Ioannina, GRC

**Keywords:** biopsychosocial model, daytime napping, dream recall, humor styles, loneliness, older adults, sleep quality

## Abstract

Objectives: Sleep experiences in later life are shaped by multiple biological, psychological, and social factors. This exploratory cross-sectional study examined associations between subjective sleep quality, daytime napping, dream recall, and selected health and psychosocial variables in community-dwelling older adults, within a biopsychosocial framework.

Methods: A cross-sectional questionnaire-based survey was conducted in Greece among 609 community-dwelling adults aged 60 years and older. Participants completed self-report measures of health-related quality of life (SF-36), loneliness, humor styles, and brief single-item measures of subjective sleep quality, sleep sufficiency, daytime napping, and dream recall. Descriptive statistics, Spearman correlations, Mann-Whitney U tests, and multiple regression analyses were performed. Given the exploratory design and the use of multiple statistical tests without correction for multiple comparisons, the findings were interpreted cautiously.

Results: Sleep quality was positively associated with mental health (ρ=0.247), physical health (ρ=0.211), and affiliative humor (ρ=0.148) and negatively associated with emotional loneliness (ρ=-0.215). Sleep sufficiency and sleep quality were almost perfectly correlated (ρ=0.989), suggesting substantial conceptual overlap. Daytime napping was positively associated with age (ρ=0.184) and was more frequent in men than women. Dream recall was positively associated with physical health (ρ=0.141) and self-enhancing humor (ρ=0.202) and negatively associated with social loneliness (ρ=-0.136). In multivariable analyses, mental health (β=0.145; p=0.049) and affiliative humor (β=0.159; p=0.001) were independently associated with sleep quality.

Conclusion: Subjective sleep experiences in older adults showed modest associations with health status, loneliness, and humor styles. These findings should be considered preliminary and exploratory, particularly because key sleep and dream variables were assessed using non-validated single-item measures, several associations were modest in magnitude, and multiple statistical tests were conducted without correction for multiple comparisons. The results may help generate hypotheses for future studies using validated multidimensional sleep and dream measures, but they should not be interpreted as confirmatory or causal evidence.

## Introduction

Sleep in later life represents both a biological necessity and a sensitive indicator of overall health and functional integrity. Aging is accompanied by substantial changes in sleep architecture, circadian regulation, and sleep continuity, which may influence cognitive functioning, emotional regulation, and physical well-being [1]. In recent years, sleep has increasingly been conceptualized within the broader framework of sleep health, a multidimensional construct encompassing sleep duration, continuity, timing, regularity, and daytime alertness [2,3]. From this perspective, sleep quality reflects not only physiological processes but also psychological adaptation and social context. In addition, sleep quality has been linked to broader physiological regulation, including nutritional and metabolic status [4].

Sleep disturbances are highly prevalent among older adults and are associated with a wide range of adverse outcomes, including cardiometabolic disorders and reduced quality of life [5]. Poor sleep quality has been linked to increased risk of cardiovascular disease, metabolic dysregulation, cognitive decline, and reduced quality of life [2,6]. Further studies suggest that multidimensional sleep problems may predict the onset of neurodegenerative disorders, including dementia [7]. Consequently, understanding the factors that shape sleep experiences in later life has become a central priority in contemporary aging research.

Beyond nocturnal sleep itself, other dimensions of the sleep-wake cycle, particularly daytime napping and dreaming, offer valuable insights into the biopsychosocial processes underlying healthy aging. Daytime napping is a common practice in older populations and may serve both adaptive and maladaptive functions [8]. Short naps have been shown to enhance alertness, attention, and memory consolidation, particularly when nocturnal sleep is insufficient [9]. However, longer or irregular naps have been associated with increased risks of cardiovascular disease, metabolic syndrome, and cognitive impairment [10,11]. Cultural context further complicates the interpretation of napping behavior. In Mediterranean societies, for example, midday rest has historically formed part of daily rhythms, suggesting that the health implications of napping may vary across cultural environments.

Dreaming has been associated with emotional processing and the regulation of affective experiences during sleep [12,13]. Contemporary neuroscience suggests that dreams contribute to emotional processing, memory consolidation, and the integration of waking experiences [14]. At the same time, alterations in dream frequency or content have been associated with psychological distress and neurodegenerative processes. Recent longitudinal evidence indicates that frequent distressing dreams may predict future cognitive decline and dementia risk [15,16]. These findings suggest that dreaming may serve as a potential non-invasive marker of brain and psychological health in later life.

Sleep in older adults is shaped not only by biological processes but also by psychological and social factors, consistent with the biopsychosocial model of health. Loneliness has been identified as an important correlate of sleep disturbance, with perceived social isolation associated with sleep fragmentation and reduced sleep quality [17]. Such effects may be mediated by increased physiological arousal and stress responses [18]. In contrast, psychosocial resources such as positive affect, resilience, and adaptive coping may support more favorable sleep experiences.

Humor represents one such psychosocial resource. According to the humor styles model, affiliative and self-enhancing humor are considered more adaptive forms associated with social connection and emotional regulation, whereas aggressive and self-defeating humor reflect less adaptive patterns [19]. Through its role in modulating emotional tension and facilitating interpersonal engagement, humor may be associated with sleep experiences. However, empirical evidence on the relationship between humor styles and sleep remains limited.

Although sleep in older adults has received increasing attention, much of the available evidence has focused on single dimensions such as sleep quality or daytime napping rather than examining sleep quality, napping, and dream-related experiences simultaneously within a broader psychosocial framework. This appears to be particularly true in Mediterranean research, where available studies have primarily addressed isolated aspects of sleep-related behavior [20,21].

The present study was guided by a biopsychosocial framework, which conceptualizes sleep as the outcome of interacting biological, psychological, and social processes. Within this perspective, we explored associations between subjective sleep quality, daytime napping, and dream recall and selected biopsychosocial variables, including health status, loneliness, and humor styles, in a sample of community-dwelling older adults.

Sleep quality was considered the primary outcome, while daytime napping and dream recall were examined as secondary, complementary dimensions of sleep experience. Given the limited prior evidence, particularly regarding dream-related outcomes and humor styles, and the use of brief self-report indicators for key sleep and dream variables, the analyses were primarily exploratory in nature and were not guided by specific directional hypotheses.

## Materials and methods

Study design and participants

This study employed a cross-sectional design to investigate the associations between sleep experiences, health status, loneliness, and humor styles among older adults.

Participants were 609 community-dwelling older adults in Greece, aged 60 years and older. Individuals were recruited through community centers, local health units, and social associations. Eligibility criteria included the following: (a) age ≥60 years, (b) ability to complete the questionnaire independently, and (c) absence of severe cognitive impairment that could interfere with participation.

Participation was voluntary and based on informed consent. All responses were anonymized prior to analysis.

Ethical approval for the study was obtained from the Ethics Committee of the University of Ioannina (approval number: 27943; date: July 24, 2024). All procedures were conducted in accordance with the principles of the Declaration of Helsinki and the General Data Protection Regulation (GDPR). Participants provided electronic informed consent prior to participation, were assured of anonymity and confidentiality, and retained the right to withdraw from the study at any time without consequence. No sensitive personal data was collected.

Measures

Sociodemographic and Social Characteristics

Participants completed a structured questionnaire assessing demographic and social characteristics, including age, gender, height, weight, marital status, educational level, number of siblings and children, occupational status, and place of residence. Additional items assessed perceived social support in daily life, including the availability of instrumental and emotional assistance.

Health-Related Quality of Life

Health status was assessed using the Short Form Health Survey (SF-36) [22]. The instrument evaluates eight domains of health-related quality of life: Physical Functioning (PF), Role Physical (RP), Bodily Pain (BP), General Health (GH), Vitality (VT), Social Functioning (SF), Role Emotional (RE), and Mental Health (MH). Each domain is scored from 0 to 100, with higher scores indicating better perceived health.

Following standard procedures, subscales were summarized into the Physical Component Summary (PCS-36) and Mental Component Summary (MCS-36) [23]. In the present sample, internal consistency of the SF-36 components was satisfactory (Cronbach's α=0.888).

Sleep and Dream Variables

Subjective sleep experience was assessed using five brief self-report items capturing key aspects of sleep and dream experience: perceived sufficiency of sleep, perceived sleep quality, frequency of daytime napping, dream recall frequency, and attitudes toward remembering dreams.

Items assessing sleep sufficiency, sleep quality, daytime napping, and dream recall were rated on 5-point Likert scales (1=very poor/never to 5=very good/always), while attitudes toward remembering dreams were assessed on a 3-point scale (no, indifferent, yes). Higher scores indicated a greater presence of the respective attribute.

These items were developed for the purposes of the present study to provide a brief and feasible assessment of subjective sleep and dream experiences in community-based older adults. As they were not part of a previously validated standalone questionnaire, they should be interpreted as brief subjective indicators rather than comprehensive or psychometrically validated measures. Consequently, they may be more susceptible to measurement error and limited construct validity compared to standardized multi-item instruments.

Despite these limitations, similar single-item or brief measures have been widely used in population-based aging research as pragmatic indicators of subjective sleep experience [2,3].

Sleep and dream experiences were assessed using five author-developed self-report items created for this study to provide a brief and feasible assessment in community-based older adults. The full list of items and response formats is provided in the Appendices. Because these items were not part of a previously validated standalone questionnaire, they should be interpreted as brief subjective indicators rather than comprehensive or psychometrically validated measures of sleep and dream processes. As such, they may be more susceptible to measurement error and limited construct validity and reliability compared to standardized multi-item instruments.

Loneliness

Loneliness was assessed using the De Jong Gierveld Loneliness Scale [24]. The short six-item version captures two dimensions: emotional loneliness (absence of intimate attachment) and social loneliness (lack of a broader social network). Subscale scores were calculated according to standard scoring procedures, with higher scores indicating greater loneliness. The scale has been widely used in aging research and demonstrates satisfactory psychometric properties. In the present sample, internal consistency was acceptable (Cronbach's α=0.80).

Humor Styles

Humor styles were assessed using the Humor Styles Questionnaire (HSQ) [19], a widely used and validated instrument that measures four humor styles: affiliative humor, self-enhancing humor, aggressive humor, and self-defeating humor. Affiliative and self-enhancing humor are considered more adaptive forms of humor associated with positive emotional regulation and social functioning, whereas aggressive and self-defeating humor are typically regarded as less adaptive styles. Items are rated on Likert scales and summed to produce subscale scores, with higher values indicating stronger endorsement of each humor style. In the present sample, internal consistency was acceptable (Cronbach's α=0.76).

Procedure

Participants completed the questionnaires either independently or, when necessary, with assistance from trained research staff. Data collection took place in community settings, and all responses were recorded anonymously. Following data collection, questionnaires were coded and entered into a secure database for statistical analysis. All data were handled in accordance with confidentiality requirements and anonymized prior to analysis.

Statistical analysis

Statistical analyses were conducted using Python (Python Software Foundation, Wilmington, Delaware, United States), employing standard scientific libraries including pandas, SciPy, and statsmodels. Data preparation and initial data management were performed using spreadsheet-based tools.

Descriptive statistics (means, standard deviations, ranges, frequencies, and percentages) were calculated to summarize the demographic, health-related, and psychosocial characteristics of the sample.

Associations between sleep- and dream-related variables and biopsychosocial characteristics were examined using Spearman's rank correlation coefficients, given the ordinal nature of the Likert-type measures. Gender differences in sleep-related variables were assessed using Mann-Whitney U tests, with Cliff's delta reported as a measure of effect size.

Separate regression models were constructed for three dependent variables: sleep quality, daytime napping, and dream recall. Predictor variables included biological factors (age, gender, physical and mental health), psychological factors (loneliness), and psychosocial characteristics (humor styles). Likert-scale variables were treated as continuous in regression analyses, as is common practice when scales include five or more response categories and approximate interval properties.

All predictors were standardized prior to modeling, and variance inflation factors (VIF) were examined to assess potential multicollinearity. Missing data were low across variables (generally <3%), with the highest missingness observed for weight (n=594/609). Analyses were conducted using available cases for each variable, and listwise deletion was applied in regression models. Statistical significance was evaluated using two-tailed tests with α=0.05.

Given the exploratory nature of the analyses, no formal correction for multiple testing was applied. Therefore, the findings should be interpreted with caution, considering the increased risk of type I error.

## Results

Sample characteristics

The final sample consisted of 609 community-dwelling older adults aged 60 years and above (M=77.58; SD=8.55; range=60-99). Participants completed questionnaires assessing demographic characteristics, health-related quality of life, loneliness, humor styles, and sleep-related experiences.

Gender data were available for 608 participants, of whom 222 were men and 386 were women; one participant had missing gender data. Mean scores indicated perceived sleep quality of 3.81 (SD=1.08), daytime napping of 3.63 (SD=1.20), and dream recall of 3.00 (SD=1.12).

Health-related quality of life scores showed variability in both physical and mental health status (physical health: M=220.83 and SD=103.93; mental health: M=237.41 and SD=81.35). Mean levels of emotional loneliness (M=2.81; SD=2.19) and social loneliness (M=2.20; SD=1.50) were also observed.

Descriptive statistics for all study variables are presented in Table 1, while additional details for sleep and dream variables are provided in Table 2.

**Table 1:** Descriptive statistics of all key variables Categorical variables (gender) are presented as frequencies and percentages. Good sleep, daytime nap, and dreams were assessed on a 5-point Likert scale (1=very poor/never; 5=very good/very frequent). N: number of valid responses per variable; Mean: average; SD: standard deviation

Variable	N	Mean	SD	Min	Max
Gender					
Men	222 (36.5%)	-	-	-	-
Women	386 (63.4%)	-	-	-	-
Missing	1 (0.1%)	-	-	-	-
Continuous variables					
Age	608	77.58	8.55	60.00	99.00
Height	607	163.32	14.16	-	-
Weight	594	72.25	12.63	-	-
Physical health	608	220.83	103.93	0.00	395.83
Mental health	608	237.41	81.35	49.00	375.00
Enough sleep	608	3.81	1.09	1.00	5.00
Good sleep	608	3.81	1.08	1.00	5.00
Daytime nap	608	3.63	1.20	1.00	5.00
Dreams	608	3.00	1.12	1.00	5.00
Like remembering dreams	608	1.98	0.81	1.00	3.00
Emotional loneliness	608	2.81	2.19	0.00	6.00
Social loneliness	608	2.20	1.50	0.00	5.00
Affiliative humor	604	33.35	5.24	14.00	53.00
Self-enhancing humor	604	34.53	8.72	8.00	56.00
Aggressive humor	604	24.91	7.72	8.00	48.00
Self-defeating humor	604	26.26	7.81	8.00	51.00

**Table 2:** Descriptive statistics of sleep and dream variables All sleep/dream variables were rated on a 5-point Likert scale (1=very poor/never; 5=very good/always). "Like remembering dreams" was coded as 1=no, 2=indifferent, and 3=yes. N: number of valid responses per variable; Mean: average; SD: standard deviation

Variable	N	Mean	SD	Min	Max
Enough sleep	608	3.81	1.09	1.00	5.00
Good sleep	608	3.81	1.08	1.00	5.00
Daytime nap	608	3.63	1.20	1.00	5.00
Dreams	608	3.00	1.12	1.00	5.00
Like remembering dreams	608	1.98	0.81	1.00	3.00

Associations between sleep variables and health indicators

Correlation analyses (Table 3) indicated that sleep-related variables were associated with several indicators of physical and mental health. Perceived sleep sufficiency and overall sleep quality were very strongly correlated (ρ=0.989), indicating substantial conceptual overlap between these variables.

**Table 3:** Spearman's correlation matrix for sleep, health, and psychosocial variables Values represent Spearman's rho (ρ). Statistical significance levels are reported in the supplementary p-value matrix.

Variable	Enough_Sleep	Good_Sleep	Daytime_Nap	Dreams	Like_Dreams	Age	Height	Weight	Physical_Health	Mental_Health	Emotional_Loneliness	Social_Loneliness	Affiliative_Humor	Self_Enhancing_Humor	Aggressive_Humor	Self_Defeating_Humor
Enough_Sleep	1.000	0.989	0.321	0.178	0.181	0.020	0.103	0.032	0.215	0.250	-0.221	-0.075	0.158	0.116	-0.018	-0.022
Good_Sleep	0.989	1.000	0.313	0.178	0.182	0.019	0.108	0.037	0.211	0.247	-0.215	-0.084	0.148	0.112	-0.014	-0.012
Daytime_Nap	0.321	0.313	1.000	0.123	0.137	0.184	0.215	0.219	-0.068	-0.012	-0.021	-0.076	0.008	-0.051	0.079	-0.036
Dreams	0.178	0.178	0.123	1.000	0.976	-0.027	0.022	0.019	0.141	0.089	-0.036	-0.136	0.025	0.202	0.028	0.060
Like_Dreams	0.181	0.182	0.137	0.976	1.000	-0.004	0.002	0.008	0.111	0.070	-0.022	-0.121	0.023	0.193	0.000	0.046
Age	0.020	0.019	0.184	-0.027	-0.004	1.000	-0.090	-0.088	-0.366	-0.257	0.283	0.047	-0.150	-0.160	-0.033	-0.067
Height	0.103	0.108	0.215	0.022	0.002	-0.090	1.000	0.570	0.183	0.137	-0.183	-0.041	0.098	0.068	0.028	0.024
Weight	0.032	0.037	0.219	0.019	0.008	-0.088	0.570	1.000	-0.002	0.034	-0.122	-0.028	0.050	0.015	0.048	-0.039
Physical_Health	0.215	0.211	-0.068	0.141	0.111	-0.366	0.183	-0.002	1.000	0.785	-0.546	-0.132	0.146	0.334	-0.053	0.023
Mental_Health	0.250	0.247	-0.012	0.089	0.070	-0.257	0.137	0.034	0.785	1.000	-0.604	-0.181	0.198	0.341	-0.098	-0.004
Emotional_Loneliness	-0.221	-0.215	-0.021	-0.036	-0.022	0.283	-0.183	-0.122	-0.546	-0.604	1.000	0.128	-0.238	-0.380	0.115	0.034
Social_Loneliness	-0.075	-0.084	-0.076	-0.136	-0.121	0.047	-0.041	-0.028	-0.132	-0.181	0.128	1.000	-0.140	-0.179	0.052	-0.079
Affiliative_Humor	0.158	0.148	0.008	0.025	0.023	-0.150	0.098	0.050	0.146	0.198	-0.238	-0.140	1.000	0.398	-0.020	0.277
Self_Enhancing_Humor	0.116	0.112	-0.051	0.202	0.193	-0.160	0.068	0.015	0.334	0.341	-0.380	-0.179	0.398	1.000	0.060	0.292
Aggressive_Humor	-0.018	-0.014	0.079	0.028	0.000	-0.033	0.028	0.048	-0.053	-0.098	0.115	0.052	-0.020	0.060	1.000	0.324
Self_Defeating_Humor	-0.022	-0.012	-0.036	0.060	0.046	-0.067	0.024	-0.039	0.023	-0.004	0.034	-0.079	0.277	0.292	0.324	1.000

Sleep quality was positively associated with mental health (ρ=0.247) and physical health (ρ=0.211) and negatively associated with emotional loneliness (ρ=-0.215). Daytime napping was positively associated with age (ρ=0.184) and was more frequent among men compared to women (p<0.001; Cliff's δ=-0.355). Dream recall was positively associated with physical health (ρ=0.141) and self-enhancing humor (ρ=0.202) and negatively associated with social loneliness (ρ=-0.136).

Loneliness and sleep experiences

Loneliness was associated with sleep-related variables. Emotional loneliness was negatively associated with sleep quality (ρ=-0.215), while social loneliness showed weaker negative associations with sleep quality (ρ=-0.084) and dream recall (ρ=-0.136).

Humor styles and sleep variables

Affiliative humor was positively associated with sleep quality (ρ=0.148), while self-enhancing humor was positively associated with dream recall (ρ=0.202). Aggressive and self-defeating humor showed weaker associations with sleep-related variables, most of which were not statistically significant.

Gender differences in sleep experiences

Group comparisons (Table 4) indicated that men reported higher levels of sleep quality compared to women (M=3.97 vs. M=3.72; p=0.009; Cliff's δ=-0.121). Men also reported more frequent daytime napping (M=4.10 vs. M=3.36; p<0.001; Cliff's δ=-0.355).

**Table 4:** Gender differences in sleep and dream variables U: Mann-Whitney statistic; p: significance level; Cliff's δ: effect size

Variable	U	p	Cliff's δ	Male mean	Female mean	N (M/F)
Enough_Sleep	47923.0	0.011	-0.118	3.97	3.73	222/386
Good_Sleep	48048.5	0.009	-0.121	3.97	3.72	222/386
Daytime_Nap	58067.5	<0.001	-0.355	4.10	3.36	222/386
Dreams	37552.5	0.009	0.124	2.85	3.09	222/386
Like_Dreams	37240.0	0.004	0.131	1.86	2.05	222/386

In contrast, women reported higher levels of dream recall (M=3.09 vs. M=2.85; p=0.009; Cliff's δ=0.124) and greater preference for remembering dreams (M=2.05 vs. M=1.86; p=0.004; Cliff's δ=0.131).

Factors associated with sleep quality

Multiple regression analyses (Tables 5-7) were conducted to examine factors associated with sleep quality, daytime napping, and dream recall among older adults. Sleep sufficiency was not included as a predictor in models where sleep quality was examined as an outcome, in order to avoid multicollinearity. Results for sleep quality are presented in Table 5, for daytime napping in Table 6, and for dream recall in Table 7.

**Table 5:** Multiple regression analysis of factors associated with sleep quality β: standardized regression coefficient; CI: confidence interval

Predictor	β	p	95% CI low	95% CI high
const	3.809	<0.001	3.726	3.892
Age	0.119	0.011	0.027	0.210
Gender	-0.091	0.033	-0.174	-0.007
Physical_Health	0.080	0.269	-0.063	0.224
Mental_Health	0.145	0.049	0.000	0.290
Emotional_Loneliness	-0.078	0.163	-0.188	0.032
Social_Loneliness	-0.049	0.260	-0.135	0.037
Affiliative_Humor	0.159	0.001	0.066	0.252
Self_Enhancing_Humor	-0.009	0.859	-0.111	0.093
Aggressive_Humor	0.023	0.604	-0.065	0.112
Self_Defeating_Humor	-0.060	0.218	-0.154	0.035

**Table 6:** Multiple regression analysis of factors associated with daytime napping β: standardized regression coefficient; CI: confidence interval

Predictor	β	p	95% CI low	95% CI high
const	3.632	<0.001	3.543	3.721
Age	0.208	<0.001	0.109	0.307
Gender	-0.345	<0.001	-0.435	-0.255
Physical_Health	-0.138	0.079	-0.292	0.016
Mental_Health	0.077	0.335	-0.080	0.233
Emotional_Loneliness	-0.084	0.162	-0.202	0.034
Social_Loneliness	-0.102	0.031	-0.195	-0.009
Affiliative_Humor	0.110	0.033	0.009	0.210
Self_Enhancing_Humor	-0.107	0.055	-0.218	0.003
Aggressive_Humor	0.097	0.046	0.002	0.192
Self_Defeating_Humor	-0.054	0.303	-0.156	0.049

**Table 7:** Multiple regression analysis of factors associated with dream recall β: standardized regression coefficient; CI: confidence interval

Predictor	β	p	95% CI low	95% CI high
const	3.003	<0.001	2.917	3.089
Age	0.022	0.643	-0.073	0.118
Gender	0.130	0.003	0.043	0.217
Physical_Health	0.210	0.005	0.062	0.358
Mental_Health	-0.074	0.336	-0.224	0.077
Emotional_Loneliness	0.094	0.103	-0.019	0.208
Social_Loneliness	-0.121	0.008	-0.211	-0.032
Affiliative_Humor	-0.072	0.143	-0.169	0.024
Self_Enhancing_Humor	0.217	<0.001	0.111	0.323
Aggressive_Humor	0.032	0.487	-0.059	0.124
Self_Defeating_Humor	-0.000	0.997	-0.098	0.098

Mental health (β=0.145; p=0.049) and affiliative humor (β=0.159; p=0.001) were positively associated with sleep quality. Age (β=0.119; p=0.011) was also positively associated, while gender (β=-0.091; p=0.033) indicated that women reported lower sleep quality compared to men. The variance explained by the models was modest (R² ranging from 0.11 to 0.18).

Physical health, emotional loneliness, social loneliness, and the remaining humor styles were not statistically significant predictors in the multivariable model. 

## Discussion

Integrating sleep quality in later life

The present findings indicate that sleep quality in later life is associated with broader biopsychosocial functioning rather than representing a simple byproduct of aging. Although several associations reached statistical significance, their magnitude was generally small, suggesting that these relationships should be interpreted with caution. Consistent with previous research, better perceived mental and physical health were associated with improved subjective sleep quality [10], supporting the view that sleep quality reflects the interplay of physiological and psychological processes.

Affiliative humor was also positively associated with sleep quality, highlighting the potential relevance of positive affect and social interaction. Prior research suggests that affiliative humor may buffer stress and reduce physiological arousal through social bonding [19]. In this context, humor may be considered a psychosocial resource associated with emotional regulation and more favorable sleep experiences.

Taken together, these findings are consistent with a perspective in which sleep quality is associated with a network of interacting biopsychosocial factors rather than determined by a single dominant mechanism.

Daytime napping

Daytime napping was associated with age and gender, with older adults and men reporting more frequent napping. This pattern is consistent with previous research indicating that napping tends to increase with advancing age, potentially reflecting changes in sleep continuity.

Previous studies suggest that short naps may enhance alertness and cognitive performance [9], whereas prolonged or irregular napping has been linked to adverse outcomes, including cardiometabolic risk and cognitive decline [11]. In the present study, napping was also associated with selected psychosocial characteristics, suggesting that daytime sleep behavior may reflect not only physiological needs but also broader behavioral patterns.

Dream recall and its psychological correlates

Dream recall was positively associated with physical health, self-enhancing humor, and female gender. These findings are consistent with previous literature linking dream recall to cognitive activation and emotional processing during sleep [25].

The association between self-enhancing humor and dream recall may reflect, tentatively, a link with cognitive and emotional processing, although this interpretation remains speculative. Similarly, lower dream recall in individuals with higher social loneliness may be cautiously interpreted as reflecting reduced engagement with socially meaningful experiences, which are often represented in dream content [15].

Additionally, alterations in dream recall have been linked to neurocognitive processes and may represent potential early indicators of neurological vulnerability [16]. Although the present study does not examine longitudinal outcomes, these findings highlight the possible relevance of dream experiences within broader models of psychological and cognitive functioning.

Psychological and emotional regulation

The findings are consistent with broader frameworks of emotional regulation. The association between affiliative humor and sleep quality aligns with theories emphasizing the role of positive emotions in adaptive coping and emotional regulation [26]. Similarly, self-enhancing humor may reflect a cognitive style associated with resilience and psychological stability.

The observed relationship between loneliness and sleep is consistent with models suggesting that perceived social isolation may increase physiological arousal and vigilance [27]. Together, these findings suggest that psychosocial characteristics are associated with sleep experiences in later life.

Interpretive perspective

From an interpretive perspective, humor may be viewed as a mature regulatory process, although this interpretation should be considered theoretical and hypothesis-generating [28]. In this context, affiliative and self-enhancing humor may be associated with greater capacity for emotional regulation.

Similarly, the observed associations between dream recall, health, and loneliness may be interpreted within broader frameworks emphasizing the role of symbolic and relational processing [29-31]. These interpretations remain speculative and are presented as potential avenues for future research rather than as empirically established explanations.

Toward a biopsychosocial perspective

The findings of the present study support an integrative biopsychosocial perspective in which sleep experiences in later life are associated with biological health, psychological functioning, and social context. Importantly, these findings reflect associations observed within a cross-sectional design and do not imply causal relationships.

The modest variance explained by the models suggests that a substantial proportion of variability in sleep-related outcomes remains unaccounted for, indicating that additional determinants of sleep may not have been captured in the present study.

While the present study adopts a biopsychosocial framework, it does not examine specific mechanisms or structured pathways. Future research may build on these findings by investigating such processes using longitudinal and methodologically rigorous designs.

Clinical and public health implications

From a clinical perspective, the findings should be interpreted cautiously, as the observed associations were modest and derived from a non-clinical, community-based sample. Therefore, they do not imply direct clinical applications.

In addition to established approaches such as cognitive behavioral therapy for insomnia, interventions that promote social engagement, emotional well-being, and adaptive coping may warrant further investigation. Daytime napping patterns may also represent clinically relevant indicators, as excessive napping could reflect underlying dysregulation.

At the public health level, integrating sleep promotion with interventions targeting loneliness and social participation may contribute to healthier aging. Any potential clinical implications should be considered indirect and hypothesis-generating, requiring confirmation in longitudinal and clinical studies.

Strengths and limitations

The study has several strengths, including a large community-based sample and the simultaneous examination of multiple dimensions of sleep experience within a biopsychosocial framework. However, several limitations should be acknowledged.

First, the cross-sectional design precludes any inference regarding causality or temporal direction. The observed associations may reflect bidirectional or confounded relationships, and longitudinal or experimental studies are required to clarify potential causal pathways.

Second, the recruitment strategy, which relied on community-based settings such as social centers and local associations, may have introduced selection bias. Participants were likely to be more socially active and functionally independent than more isolated or clinically vulnerable older adults, limiting the generalizability of the findings.

Third, all variables were assessed using self-report measures, which may introduce common method bias and inflate observed associations due to shared variance and response tendencies.

Fourth, the assessment of sleep and dream variables relied on brief, author-developed single-item measures that have not been formally validated. This represents an important limitation, as it may reduce measurement precision and limit the construct validity of key variables.

Fifth, the extremely high correlation between sleep sufficiency and sleep quality suggests substantial conceptual overlap and limited discriminant validity, indicating that participants may have perceived these variables as closely related aspects of subjective sleep experience.

Sixth, no correction for multiple comparisons was applied, increasing the risk of type I error. Therefore, some statistically significant findings may reflect chance associations.

Finally, the observed associations were generally modest in magnitude, and the regression models explained a limited proportion of variance. These findings should be interpreted with caution, as they likely reflect subtle relationships within a complex biopsychosocial system rather than strong or clinically meaningful effects.

## Conclusions

The present study provides exploratory evidence that sleep experiences in later life are associated with a range of biological, psychological, and social factors within a biopsychosocial framework. These associations were generally modest and derived from non-clinical, self-reported data and should therefore be interpreted with caution.

The findings underscore the relevance of considering psychosocial dimensions alongside physical health in understanding sleep in older adults while also indicating that important determinants of sleep outcomes may not have been fully captured in the present models.

Future research should prioritize the use of validated measures, longitudinal designs, and more diverse and clinical populations to clarify underlying mechanisms. In particular, further investigation in clinical and psychiatric contexts may help determine whether psychosocial factors such as loneliness and humor styles have clinically meaningful relevance for sleep disturbances.

Overall, this study contributes by adopting an integrative biopsychosocial perspective and identifying patterns that may inform more targeted and theory-driven research in this field.

## Appendices

**Table 8:** Items assessing sleep and dream experiences

Item	Question	Response scale
1	How sufficient do you consider your sleep?	1=very insufficient to 5=very sufficient
2	How would you rate your overall sleep quality?	1=very poor to 5=very good
3	How often do you nap during the day?	1=never to 5=very often
4	How often do you remember your dreams?	1=never to 5=very often
5	Do you like remembering your dreams?	1=no; 2=indifferent; 3=yes
